# Effect of Substrate on Sulfur Vacancy Defect-Mediated Photoluminescence in Two-Dimensional MoS_2_

**DOI:** 10.1021/acs.jpcc.4c08491

**Published:** 2025-04-18

**Authors:** Yiru Zhu, Zhepeng Zhang, Ye Wang, Soumya Sarkar, Yang Li, Han Yan, Larissa Ishibe-Veiga, Anita Bagri, Ziwei Jeffrey Yang, Hugh Ramsden, Goki Eda, Robert L.Z. Hoye, Yan Wang, Manish Chhowalla

**Affiliations:** 1 Department of Materials Science and Metallurgy, University of Cambridge, 27 Charles Babbage Rd, Cambridge CB3 0FS, U.K.; 2 Department of Physics, National University of Singapore, 2 Science Drive 3, Singapore 117551, Singapore; 3 Diamond Light Source, Chilton, Didcot, Oxfordshire OX11 0DE, U.K.; 4 Department of Chemistry, National University of Singapore, 3 Science Drive 3, Singapore 117543, Singapore; 5 Centre for Advanced 2D Materials, National University of Singapore, 6 Science Drive 2, Singapore 117542, Singapore; 6 Inorganic Chemistry Laboratory, University of Oxford, South Parks Road, Oxford OX1 3QR, U.K.

## Abstract

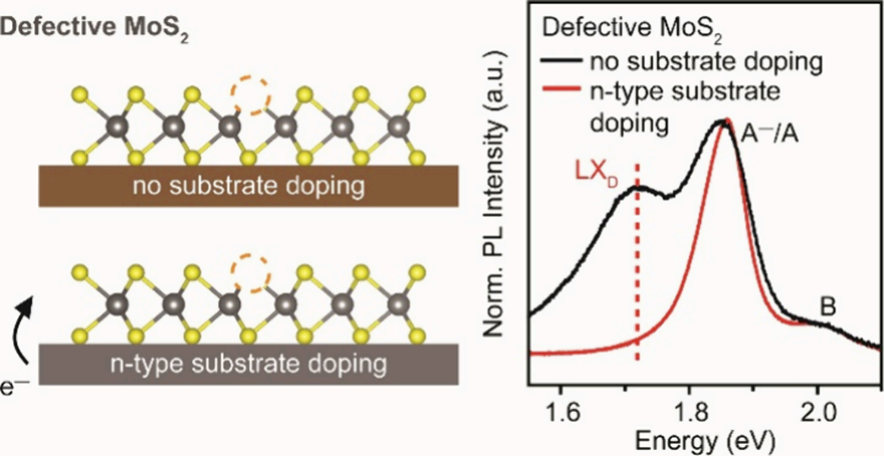

Chalcogen vacancy defects in monolayer transition metal dichalcogenides form in-gap states that can trap excitons, leading to defect-mediated photoluminescence (PL) emission. Here, we show that room-temperature (RT, 300 K) PL from sulfur vacancies in defective monolayer MoS_2_ is sensitive to doping from dielectric substrates such as SiO_2_ and HfO_2_. The defect-mediated PL is observed for monolayer MoS_2_ on untreated HfO_2_ but is quenched on untreated SiO_2_, which is attributed to electron doping of MoS_2_ on SiO_2_. Electron doping of MoS_2_ is confirmed by Raman and synchrotron X-ray photoelectron spectroscopy. Annealing of the SiO_2_ substrate modifies its surface states, which is reflected in the recovery of the defect-mediated PL emission. The role of substrate-induced doping on sulfur vacancy-mediated PL is further supported by gate-dependent PL measurements. Our results suggest that excess electrons fill the defect energy states from sulfur vacancies in MoS_2_, reducing the probability of photoexcited carrier occupation and subsequent defect-mediated emission.

Defects in two-dimensional (2D) transition metal dichalcogenides (TMDs) have properties that are useful for optoelectronics,^1^ sensing,^2^ and electro/photocatalysis.^3,4^ These properties are related to defect-induced in-gap states that modify the electronic structure of the host crystal. The in-gap states can trap excitons and give rise to defect-mediated photoluminescence (PL)^5,6^ and even single-photon emission.^7,8^ Defect-engineered TMDs, such as MoS_2_ and WSe_2_, have shown the ability to host quantum emitters, but their operation is currently limited to cryogenic temperatures, typically below 150 K.^8^ This limitation highlights the need for further research to improve the thermal stability and performance of defect-related PL in TMDs, including defect engineering^9^ and external environment (e.g., substrate) engineering. Compared with defects in 3D semiconductors, defects in 2D semiconducting TMDs are subject to reduced dielectric screening that leads to enhanced Coulombic interactions. As a result, their electronic and optical properties are susceptible to external stimuli such as strain,^10−12^ dielectric screening,^13−15^ and charge transfer.^16,17^ These effects often originate from the dielectric substrates on which monolayer TMDs are placed. Although the effect of the substrate on the excitonic PL emission from monolayer TMDs at ∼1.9 eV is well studied,^18^ how the properties of the substrate influence defect-mediated PL emission has not been well explored—partially because the methods used to introduce defects to the TMDs can also modify the underlying substrate.^19^ A recent study showed that c-sapphire substrate can electron-dope MoS_2_ and quench the defect PL emission at 10 K,^20^ consistent with previous reports on the tunability of single-photon emission from atomic defects at 80 K through electrostatic gate control.^21^ However, the effect of dielectric substrates on chalcogen vacancy defects and their PL emission at room temperature is not yet clear. Understanding these effects is critical, as they could significantly influence the performance of optoelectronic devices. For instance, in light-emitting diodes (LEDs), chalcogen vacancies can influence emission characteristics, while in photodetectors and solar cells, they can affect light absorption and charge carrier dynamics, leading to tunable emission wavelengths, enhancing light–matter interactions, and optimizing charge carrier lifetimes at localized states. Such insights would not only improve the reliability of single-photon emitters and boost photovoltaic efficiency but also enable the design of optoelectronic devices with higher performance and greater energy efficiency.

We recently reported room-temperature (RT, ∼300 K) PL from sulfur vacancies that were generated by thermally annealing monolayer MoS_2_.^22^ In this work, we investigate the influence of various dielectric substrates on sulfur-vacancy-mediated PL emission in monolayer MoS_2_. This defect-mediated PL from defective MoS_2_ was observed when it was placed on untreated HfO_2_, but not on untreated SiO_2_. This is attributed to the higher electron doping effect from SiO_2_. We demonstrate modification of the surface states of dielectric substrates by annealing using synchrotron X-ray absorption spectroscopy (XAS) and water contact angle (WCA) measurements. We find that annealing SiO_2_ substrates leads to reduced electron doping in MoS_2_ so that defect-mediated PL can be observed. In contrast, annealing the HfO_2_ substrate leads to enhanced electron doping and suppression of defect-mediated PL at room temperature in MoS_2_. Our hypothesis is further supported by gate-dependent PL measurements, which show that defect-mediated PL emission is suppressed by electron doping.

## Results and Discussion

**Figure 1:**
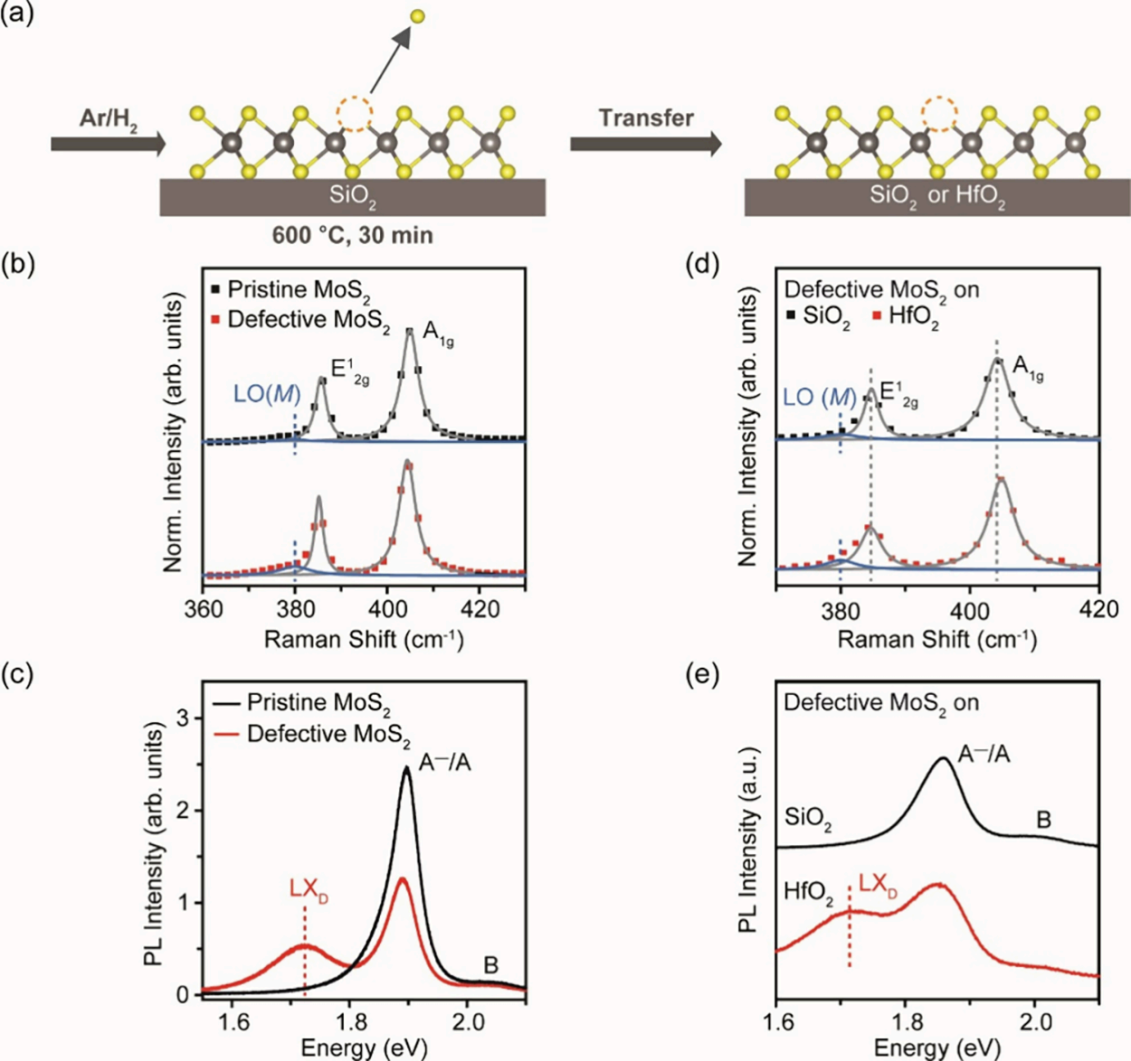
Transfer of defective monolayer MoS_2_ (Ar/H_2_ annealed) onto untreated SiO_2_ and HfO_2_, respectively. (a) Schematic of defective MoS_2_ transferred from the original SiO_2_ substrate to other substrates. (b) Raman spectra of pristine and defective MoS_2_ on original SiO_2_, normalized to the intensity of the Si reference peak. Pristine MoS_2_ shows the characteristic E^1^_2g_ in-plane mode and the A_1g_ out-of-plane mode, denoted in gray. Defective MoS_2_ shows an additional LO(*M*) phonon mode that is taken as a signature of sulfur vacancies, denoted in blue. (c) RT PL spectra of pristine and defective MoS_2_ on the original SiO_2_. Pristine MoS_2_ shows the A^–^/A peak and the B exciton peak, while defective MoS_2_ shows an additional LX_D_ emission that is mediated by sulfur vacancies. (d) Raman spectra of transferred defective monolayer MoS_2_, normalized to the intensity of Si reference peak. The LO(*M*) mode remains in transferred samples, implying that the transferred samples remain defective. (e) PL spectra of transferred defective monolayer MoS_2_. The LX_D_ peak is observed in defective MoS_2_ on HfO_2_, but not on SiO_2_.

Monolayer MoS_2_ samples on SiO_2_ (300 nm)/Si wafers were prepared by mechanical exfoliation and annealed in Ar/H_2_ (95 vol %/5 vol %) at 600 °C for 30 min, as shown in Figure 1a, left panel. Figure 1b shows the Raman spectrum of pristine monolayer MoS_2_ with the characteristic E^1^_2g_ in-plane mode at ∼385 cm^–1^ and the A_1g_ out-of-plane mode at ∼405 cm^–1^. The PL spectrum in Figure 1c shows a dominant A^–^ (negatively charged exciton)/A (neutral exciton) PL peak at ∼1.89 eV and a B exciton peak at ∼2.04 eV.^23^ For the annealed samples, the generation of sulfur vacancy defects was confirmed by the evolution of a satellite Raman peak [LO(*M*)] at ∼380 cm^–1^ (Figure 1b) and PL from sulfur vacancies (LX_D_) (Figure 1c)—consistent with our recent work.^22^ The defective MoS_2_ flake was then picked up from the SiO_2_ substrate upon which it was annealed and transferred onto untreated SiO_2_ and HfO_2_ (thickness ∼ 50 nm) substrates (Figure 1a, right panel). The untreated substrates refer to the substrates cleaned with acetone and isopropanol only without further treatment. The optimization of the transfer method is described in the Supporting Information (SI), Figures S1–S3.

The Raman spectra of transferred defective MoS_2_ show the presence of the LO(*M*) defect mode on the two substrates (untreated SiO_2_ and HfO_2_), as shown in Figure 1d. The E^1^_2g_ mode remains at ∼385 cm^–1^ while the A_1g_ mode is hardened on HfO_2_ (Δω = +0.4 cm^–1^; ω is the wavenumber of the Raman peak) than on SiO_2_, indicating that MoS_2_ contains a higher electron concentration (Δ*n* = +1.2 × 10^12^ cm^–2^; *n* is the electron concentration) on SiO_2_ than on HfO_2_.^24^ The higher electron doping from SiO_2_ than from HfO_2_ is supported by synchrotron XPS measurements (SI, Figure S4). Pristine MoS_2_ on SiO_2_ shows the characteristic Mo 3d doublet at 230.4 eV (Mo 3d_5/2_), 0.54 eV higher than that of pristine MoS_2_ on HfO_2_, implying that MoS_2_ is less electron-doped by HfO_2_. The LX_D_ peak in PL spectra (Figure 1e) however disappears when defective MoS_2_ is transferred to SiO_2_ and is observed only when defective MoS_2_ is transferred to HfO_2_.

We have also examined the impact of dielectric screening in comparing MoS_2_ on untreated HfO_2_ and SiO_2_. The dielectric environment plays a crucial role in influencing both the electronic bandgap and the exciton binding energy, which collectively determine the optical bandgap. When comparing HfO_2_ to SiO_2_, the enhanced screening effect in HfO_2_ is expected to weaken electron–electron interactions, resulting in a reduction of quasiparticle bandgap renormalization.^25^ Similarly, the electron–hole interactions are also reduced, leading to a decrease in the exciton binding energy. The overall shift in the PL depends on which of these two competing effects dominates. In our observations, the excitonic PL positions of MoS_2_ on SiO_2_ and HfO_2_ show negligible differences, suggesting that the two effects may effectively cancel each other out—consistent with density functional theory (DFT) calculations.^26^ Regarding defect-mediated PL, deep defect states arising from sulfur vacancies are positioned far from the band edges and are pinned below the conduction band edge with a fixed energy gap, as indicated by DFT calculations.^27^ This configuration reduces the binding energy of excitons to the defect states, which is expected to enhance the delocalization of trapped excitons and lead to a decrease in defect-mediated PL intensity. However, our findings reveal an opposite trend in that the defect-mediated PL intensity increases in MoS_2_ on HfO_2_ compared to MoS_2_ on SiO_2_, indicating that other factors may be at play in this system.

**Figure 2:**
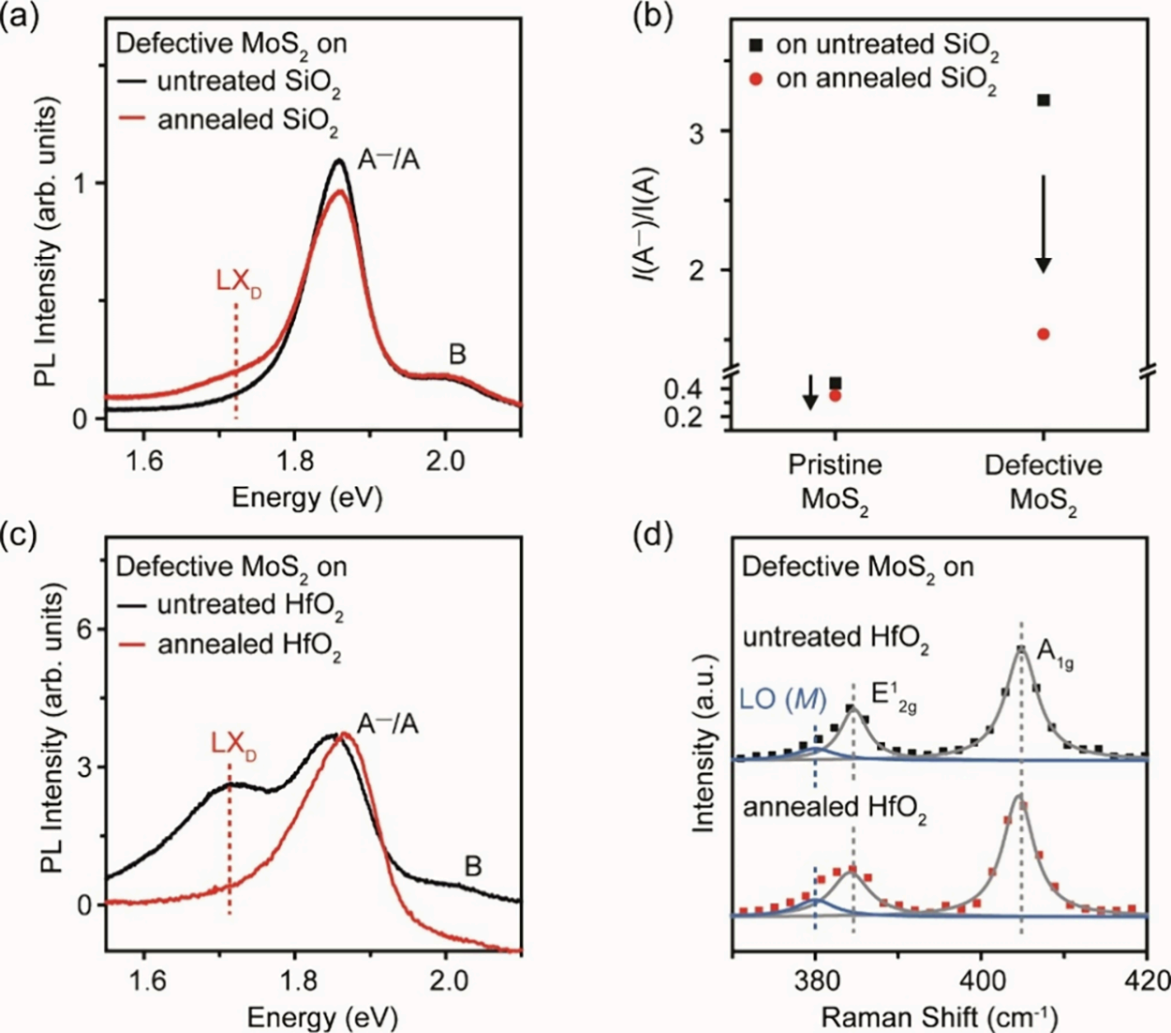
Transfer of defective monolayer MoS_2_ onto untreated and Ar/H_2_-annealed SiO_2_ (600 °C, 30 min). (a) PL spectra of transferred defective monolayer MoS_2_ on untreated and annealed SiO_2_. The LX_D_ peak is observed in defective MoS_2_ on annealed SiO_2_ but not on untreated SiO_2_. (b) Ratio of integrated intensity of the A^–^ trion to the A exciton, *I*(A^–^)/*I*(A), for pristine and defective MoS_2_ on untreated and annealed SiO_2_, respectively. For both pristine and defective MoS_2_, the *I*(A^–^)/*I*(A) ratio is lower on annealed SiO_2_ than that on untreated SiO_2_. (c) RT PL spectra of the defective monolayer MoS_2_ on HfO_2_. The defective MoS_2_ shows strong LX_D_ emission on untreated HfO_2_, but not on annealed HfO_2_. (d) RT Raman spectra of defective monolayer MoS_2_ on HfO_2_. The red shift of the A_1g_ peak is attributed to stronger electron doping of MoS_2_ on annealed HfO_2_.

To verify that the disappearance of the LX_D_ peak is related to substrate effects from SiO_2_ and not the transfer process, we picked up defective MoS_2_ from SiO_2_ and transferred it onto another SiO_2_ substrate that was separately annealed at 600 °C in Ar/H_2_. The PL spectrum of defective MoS_2_ shows a broad LX_D_ peak (Figure 2a, red curve), suggesting that annealing of SiO_2_ is important in observing the LX_D_ peak in defective MoS_2_. We prepared three samples per set to confirm our observations, as shown in SI, Figure S5. To compare the MoS_2_ on untreated SiO_2_ and on annealed SiO_2_, we analyzed the PL spectra to extract the ratio of the integrated intensity of A^–^ trion to A exciton, *I*(A^–^)/*I*(A) (SI, Figure S6).^28^ The *I*(A^–^)/*I*(A) ratio in defective MoS_2_ on untreated SiO_2_ (3.22) is higher than that on annealed SiO_2_ (1.54), indicating that doping from annealed SiO_2_ is suppressed. Similarly, pristine MoS_2_ shows a higher *I*(A^–^)/*I*(A) ratio on untreated SiO_2_ (0.44) than that on annealed SiO_2_ (0.35). In pristine MoS_2_ (Figure 2b), the *I*(A^–^)/*I*(A) ratio is much lower than in the defective MoS_2_ samples, because the presence of sulfur vacancies introduces free electrons in the MoS_2_.

We next analyze the effect of the HfO_2_ substrate on LX_D_ PL emission from defective MoS_2_ where we observe an opposite trend compared to that of MoS_2_ on SiO_2_. The LX_D_ peak can be observed when defective MoS_2_ is transferred on untreated HfO_2_. However, the LX_D_ peak disappears when the same defective flake is transferred onto a HfO_2_ substrate that is preannealed in Ar/H_2_ (600 °C, 30 min) (Figure 2c). A comparison of the Raman spectra of defective MoS_2_ on both substrates shows that the A_1g_ mode is softened (Δω = −0.6 cm^–1^) for annealed HfO_2_, indicating that MoS_2_ is more electron-doped by annealed HfO_2_ than that by untreated HfO_2_ (Figure 2d). The enhanced electron doping from annealed HfO_2_ indicated by our results is similar to the doping from the annealed AlO_*x*_ substrate in a previous work.^29^ These results suggest that the annealing process to generate sulfur vacancies in MoS_2_ also affects the surface states of the dielectric substrates, which determines the effective electron doping across the MoS_2_/dielectric interface.

We have probed the change in dielectric surface states using WCA and XAS (SI, Figures S7 and S8). Briefly, we find that the WCA increases in annealed SiO_2_ relative to untreated SiO_2_—suggesting transformation of hydrophilic silanol (Si–OH) to hydrophobic siloxane (Si–O–Si) groups that suppress electron transfer to MoS_2_ (SI, Figure S7).^30^ This indicates that the origin of this electron doping from SiO_2_ can be attributed to three possible mechanisms: (a) Lone-pair electrons on oxygen. The oxygen atom in a hydroxyl group has two lone pairs of electrons in the outer shell. These lone pairs are not involved in bonding and are relatively high in energy, making them available for donation. When MoS_2_ is placed on a hydroxyl-terminated SiO_2_ surface, the lone pairs on the oxygen atoms can interact with the conduction band of MoS_2_, effectively donating electrons and causing n-type doping. (b) Surface dipole moment. Hydroxyl groups create a local dipole moment due to the electronegativity difference between oxygen and hydrogen. The oxygen atom has a partially negative charge, while the hydrogen atom has a partially positive charge. This dipole moment can induce an electric field at the interface between SiO_2_ and MoS_2_, facilitating electron transfer from the SiO_2_ surface to the MoS_2_ layer. (c) Surface states. Hydroxyl groups may introduce surface states within the bandgap of SiO_2_. These states can act as electron donors if they are energetically aligned with the conduction band of MoS_2_. Conversely, annealing the SiO_2_ substrate at 600 °C in Ar/H_2_ may remove these groups and lower the doping effect. In contrast, the WCA in HfO_2_ decreases significantly after annealing. We attribute this behavior to the amorphous-to-polycrystalline phase transition in annealing HfO_2_, as indicated by XAS spectra and X-ray diffraction patterns (SI, Figures S8 and S9).^31,32^ The crystallization of HfO_2_ at high temperatures is often accompanied by the generation of oxygen vacancies that could act as electron donors, altering the surface chemistry and leading to the observed changes in properties.^33^

**Figure 3:**
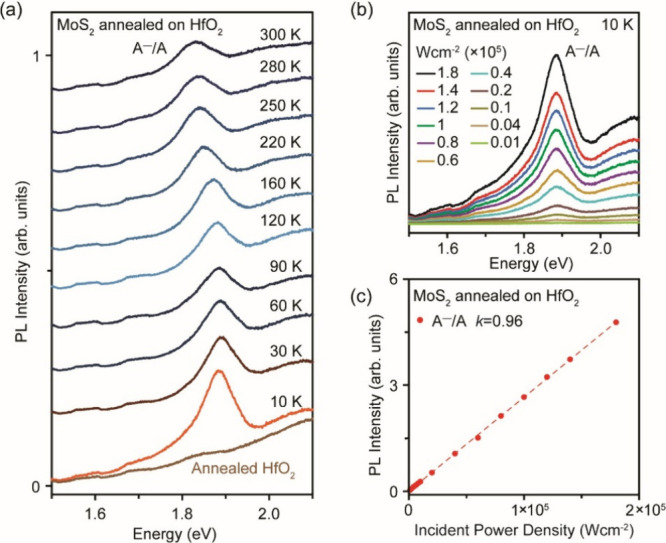
PL characterizations for MoS_2_ annealed on HfO_2_. (a) Temperature-dependent PL spectra measured at temperatures ranging from 300 to 10 K. (b) 10 K power-dependent PL spectra measured at laser fluence ranging from 0.01 to 1.8 × 10^5^ W cm^–2^. (c) Power-dependent PL intensity plot of the A^–^/A peak measured at 10 K, showing linear dependence (power exponent *k* of 0.96) that corresponds to excitonic transition.

Directly annealing monolayer MoS_2_ on HfO_2_ induces a similar suppression of the LX_D_ emission at RT (Figure S10). To confirm the suppression of LX_D_ emission on annealed HfO_2_, the PL spectrum of MoS_2_ on HfO_2_ was measured as a function of temperature ranging from 300 to 10 K (Figure 3a). Even at 10 K, the A^–^/A peak dominates over the B exciton peak (both are red-shifted by ∼55 meV) and no LX_D_ emission was observed. The PL spectra were also measured as a function of the excitation power (Figure 3b). The A^–^/A peak remains the dominant peak for laser fluence ranging from 0.01 to 1.8 × 10^5^ W cm^–2^. The intensity of the A^–^/A peak exhibits a linear dependence on excitation laser fluence (*I*_PL_ ∝ *L*_E_^*k*^) with power exponent *k* = 0.96 (Figure 3c), suggesting that the A^–^/A peak is excitonic emission that is typically associated with *k* = ∼1.^34^

**Figure 4:**
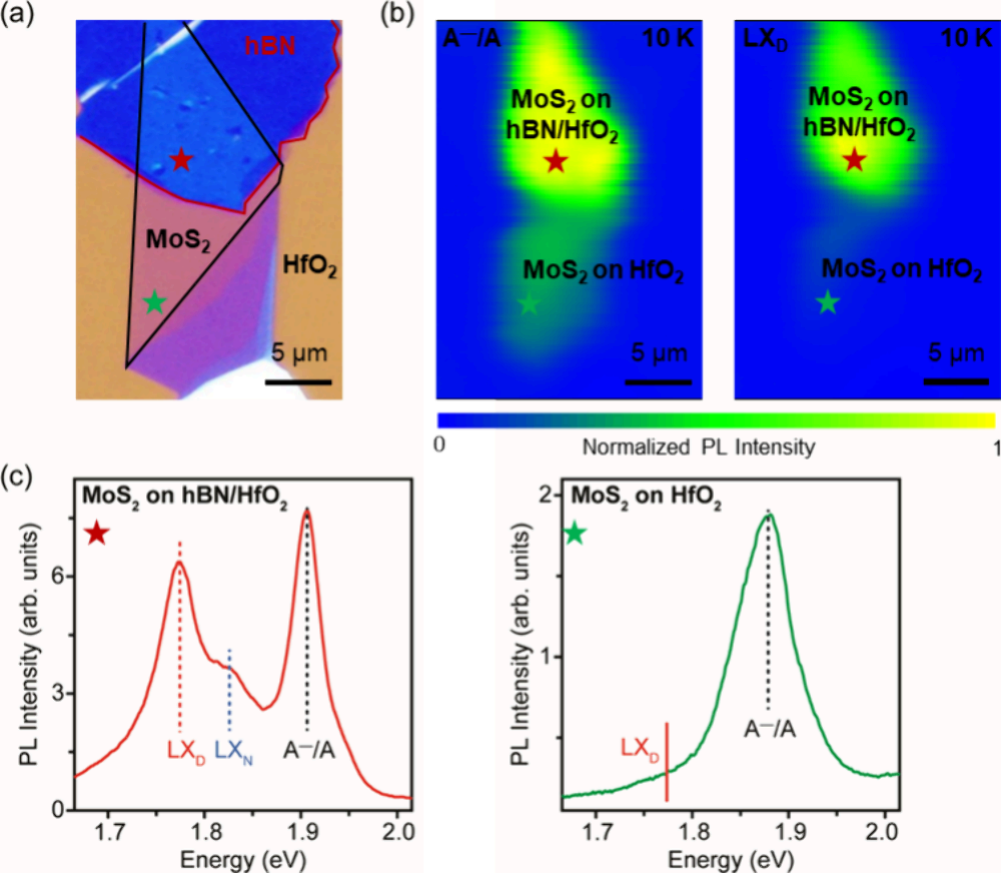
Intensity maps of A^–^/A and LX_D_ emission in Ar/H_2_ annealed (600 °C, 30 min) monolayer MoS_2_ on hBN and MoS_2_ on HfO_2_. (a) Optical microscopy images of MoS_2_ on hBN and MoS_2_ on HfO_2_. (b) Corresponding A^–^/A and LX_D_ intensity maps measured at 10 K. A^–^/A emission is distributed across MoS_2_ on hBN and MoS_2_ on HfO_2_ [left panel in (b)], whereas LX_D_ emission is only observed in MoS_2_ on hBN [right panel in (b)]. PL from two points is extracted from regions indicated by red and green stars in (a). (c) 10 K PL spectra, showing the LX_D_ peak in MoS_2_ on hBN but not in MoS_2_ on HfO_2_.

To study the PL properties of MoS_2_ without the influence of the substrate, we placed MoS_2_ on hBN. hBN is atomically flat and has low density of charged impurities.^35^ PL spectra of MoS_2_ annealed on hBN (thickness ∼ 27 nm) at 10 K are shown in Figure 4. The optical microscopy image in Figure 4a shows that MoS_2_ is partially located on HfO_2_ and hBN. In Figure 4b, the PL intensity maps of the A^–^/A peak and LX_D_ peaks measured at 10 K are shown, respectively. The A^–^/A emission was observed in both MoS_2_ on hBN and MoS_2_ on HfO_2_, whereas LX_D_ emission was only observed on MoS_2_ on hBN. Furthermore, the spectrum from MoS_2_ on hBN (indicated by the red star) and another spectrum from MoS_2_ on HfO_2_ (green star) were extracted. The LX_D_ peak is clearly visible for MoS_2_ on the hBN region but is suppressed in MoS_2_ on the HfO_2_ region (Figure 4c). This result indicates that annealed hBN does not electronically dope MoS_2_—even after annealing.

**Figure 5:**
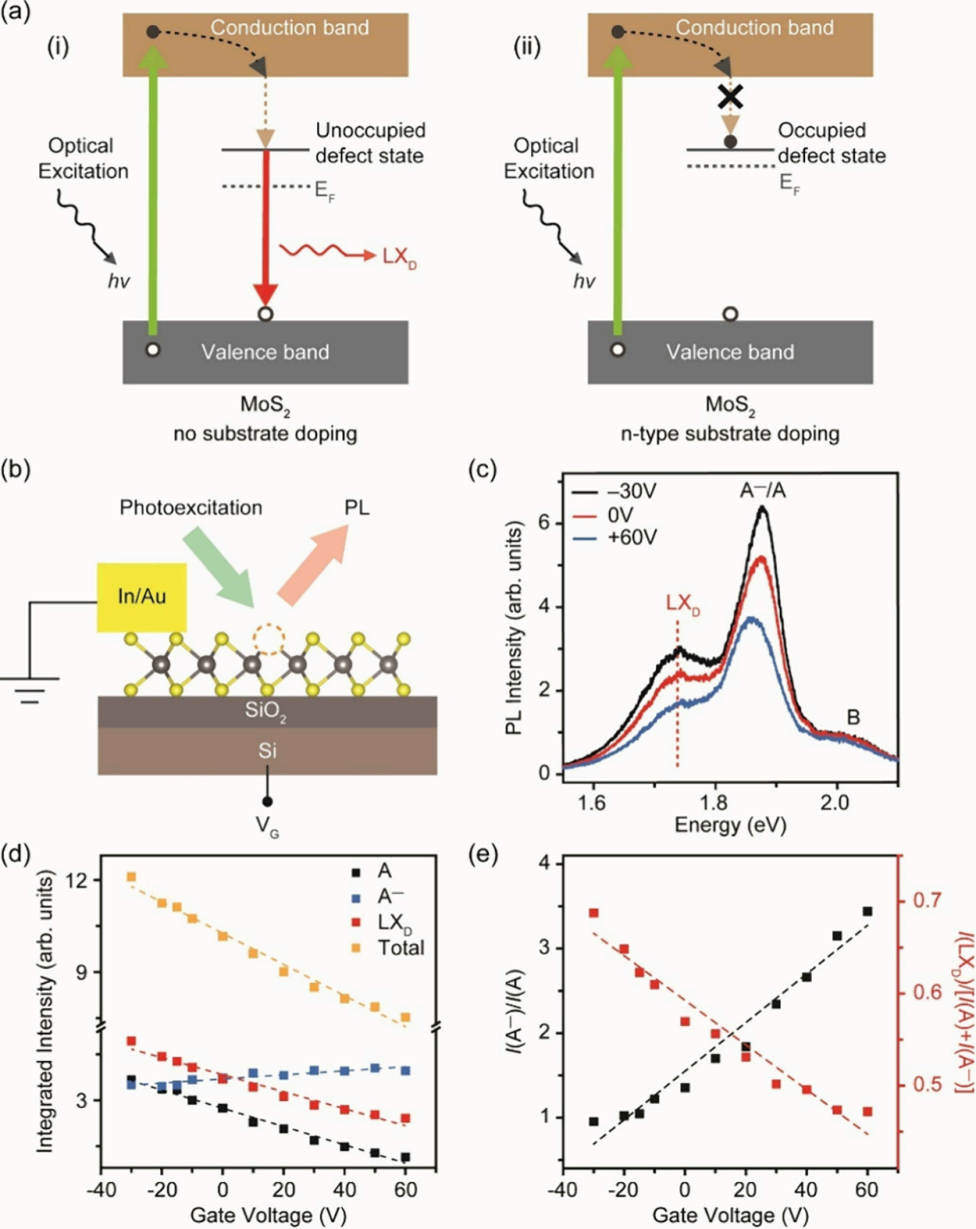
(a) Proposed schematic energy band diagram of defective monolayer MoS_2_ with different doping levels. The horizontal dashed lines represent virtual Fermi levels, while the horizontal solid line represents real energy states due to defects. An electron is photoexcited from the valence band to the conduction band (denoted by the green arrow), relaxed to the conduction band edge (denoted by the black arrow), and then relaxed to the in-gap defect state (denoted by the yellow arrow). The localized electron at the defect state can be relaxed to the valence band edge and recombine with a hole, releasing a LX_D_ photon (denoted by the red arrow). The yellow arrow represents the trapping of excitons at in-gap states. For n-typed MoS_2_ (ii), the Fermi level is higher with more valence electrons filling the in-gap states compared with undoped MoS_2_ (i), thus suppressing the trapping of photoexcited electrons and reducing the defect-mediated radiative recombination. Gate dependence of the RT LX_D_ emission. (b) Schematic of MoS_2_ FET and gate tuning of PL in defective monolayer MoS_2_. (c) RT PL spectra of defective monolayer MoS_2_ at different gate voltages (*V*_g_). (d) Integrated intensity of A exciton, A^–^ trion, and LX_D_ peak, and their total contribution. (e) Relative intensity of trions, *I*(A^–^)/*I*(A), and the integrated RT PL intensity ratio of LX_D_ emission to the sum of A exciton and A^–^ trion emission, *I*(LX_D_)/[*I*(A) + *I*(A^–^)], at different *V*_g_. At negative *V*_g_ (hole doping), the LX_D_ emission becomes relatively stronger, while at positive *V*_g_ (electron doping), the LX_D_ emission becomes relatively weaker.

The above results indicate that the LX_D_ emission is suppressed in strongly electron-doped MoS_2_. A simple energy band diagram of defective monolayer MoS_2_ with different doping levels is shown in Figure 5a. In Figure 5a(i) for the undoped MoS_2_ case, the Fermi level remains at midgap so that excited electrons can relax down to the unoccupied defect states of sulfur vacancies to form LX_D_ excitons, which can in turn radiatively recombine to emit the defect-mediated PL. In contrast, for the defective MoS_2_, electron doping from substrate can occupy the sulfur vacancy states—shifting the Fermi level closer to the conduction band and the energy of the defect states (Figure 5a(ii)).^36^ The occupation of the defect energy levels prevents relaxation of the excited electron to the defect state—preventing the formation of LX_D_ excitons and suppressing defect-mediated emission. While the preponderance of evidence suggests doping for suppression of the LX_D_ peak, other possibilities could also explain our observed results. For example, an alternative explanation for the disappearance of LX_D_ in electron-donating substrates (as-prepared SiO_2_ and annealed HfO_2_) is the short lifetime of free excitons due to rapid Auger recombination and/or the formation of free trions. That is, excitons annihilate before they have time to relax to the LX_D_ state. The occupation of the defect level may not be a necessary condition to prevent radiative recombination at LX_D_ because a defect-trapped electron can still, in principle, radiatively recombine with a hole in the valence band. We note that this model is developed based on the low laser fluence ≤17 × 10^2^ W cm^–2^ under which the photodoping effect could be negligible. The effect of doping from the substrate is similar to electrostatic doping via electrical gating—as previously reported by several groups.^28,37^ To demonstrate the relevance of gate-modulated doping for defect-mediated PL emission, we fabricated field-effect transistors (FETs) using defective monolayer MoS_2_ as the channel on 300 nm SiO_2_/Si (see the schematic in Figure 5b). It can be seen that A^–^/A decreases with increasing gate bias due to nonradiative recombination induced by the formation of trions, as observed previously by others.^38^ Here, we also find that LX_D_ emission also decreases when the channel is n-doped due to filling of defect states (Figure 5c).^38^ Deconvoluting the spectra reveals a monotonic decrease in the integrated intensities of the A exciton and LX_D_ peaks, whereas an increase in the A^–^ trion peak intensity is observed (Figure 5d). The *I*(A^–^)/*I*(A) ratio varied from 0.96 at *V*_g_ = −30 V (hole doping) to 3.44 at +60 V (electron doping).^28^ The integrated PL intensity ratio of LX_D_ emission to the sum of A exciton and A^–^ trion emission, *I*(LX_D_)/[*I*(A)+*I*(A^–^)], is inversely proportional to the *I*(A^–^)/*I*(A) ratio, confirming that electron doping reduces the defect-mediated emission LX_D_ (Figure 5e).

## Conclusions

We have shown that sulfur-vacancy-mediated PL emission from MoS_2_ depends on the electron doping level in MoS_2_. At higher doping levels, the in-gap defect states induced by sulfur vacancies are occupied by electrons, which reduces the PL emission from localized excitons. The choice of a suitable dielectric substrate is therefore essential for observation of robust LX_D_ emission. Our results suggest that MoS_2_ experiences more electron doping from the SiO_2_ substrate as compared to HfO_2_. The electron doping from the dielectric substrates originates from the presence of surface states that can be modified during the defect generation process. Our results provide useful considerations for building optoelectronic and sensing devices based on localized excitons in 2D TMDs.

## Experimental Methods

### Sample Preparation

Monolayer MoS_2_ was mechanically exfoliated from commercial bulk crystal purchased from 2D Semiconductor using a polydimethylsiloxane (PDMS)-assisted exfoliation method. Target MoS_2_ flakes were dry-transferred onto target substrate (SiO_2_ or HfO_2_) and put in a 1 in. tube furnace. The samples were flowed with 450 sccm of N_2_ (99.9999% purity) for 10 min to remove tube oxygen and then heated to 250 °C for 1 h under 100 sccm of N_2_ to clean contaminants. hBN was directly mechanically exfoliated from commercial bulk crystal purchased from HQ Graphene onto target substrates.

### Ar/H_2_ Annealing of MoS_2_

Samples were flowed with 450 sccm Ar/H_2_ (95 vol %/5 vol %) for 10 min to remove tube oxygen and heated to 200 °C under 70 sccm Ar/H_2_ for 10 min to remove tube moisture. Then, samples were heated to 600 °C and kept at that temperature for 30 min.

### ALD Growth of HfO_2_

The 50 nm HfO_2_ films were grown on doped silicon substrates by using thermal ALD in a Veeco Fiji G2 reactor. A metal alkylamide hafnium precursor tetrakis (dimethyl amido)hafnium (TDMAH) was used as the Hf source, and deionized water was the oxygen source. The reaction has been reported as follows:^39^ Hf[(CH_3_)_2_N]_4_ + 2H_2_O → HfO_2_ + 4HN(CH_3_)_2_. The TMDAH precursor was heated to 75 °C during the growth. The films were grown at a substrate temperature of 200 °C. At this temperature, the growth rate was estimated to be 1.2 Å/cycle. TDMAH and H_2_O precursors were pulsed/purged per cycle for 0.25 s/10 s and 0.06 s/10 s, respectively. 60 sccm Ar was used as the carrier gas.

### PC-Assisted Wet-Transfer Method

PC stamps were used to assist the transfer (SI, Figure S1). PC dissolved in chloroform was first drop-cast on the annealed sample to form a uniform film ①. A PDMS stamp was stacked on a glass slide and attached to the transfer stage with PDMS stamp face-down ②. The PDMS stamp was brought in contact with the PC stamp to form a firm stack ③. The PDMS/PC stack was raised to pick up the target flake from substrate 1 ④. The detached substrate 1 was replaced by another substrate 2 ⑤. The PDMS/PC stack was aligned and brought in contact with substrate 2 and heated to 180 °C for 1 min to melt PC and weaken the adhesion between PDMS and PC ⑥. The PDMS stamp was raised to detach from PC, depositing the target flake on substrate 2 ⑦. The sample was immersed in chloroform for 10 min to dissolve the melted PC stamp ⑧, leaving the target flake on substrate 2 ⑨.

### Post Annealing of Transferred MoS_2_ in Ar/H_2_

Transferred samples were flowed with 450 sccm Ar/H_2_ (95%:5%) for 10 min to remove tube oxygen and heated to 350 °C under 100 sccm Ar/H_2_ and kept for 1 h to clean PC residues.

### Measurement

- Optical images of samples were taken on a Nikon Eclipsec LV150N optical microscope.
- RT Raman and steady-state PL spectra were collected on a Renishaw inVia confocal microscope using 532 nm laser excitation. Gate-dependent PL spectra were conducted at RT on a Horiba LabRAM 800HR confocal microscope connected to a Keithley 2400 source meter with 514 nm laser excitation. The incident fluence is kept below ≤17 × 10^2^ W cm^–2^ to ensure minimal sample heating.
- Cryogenic PL spectra, PL mapping, and time-resolved PL measurements were measured on a Kymera spectrometer and Janis (ST-500) cryostat with a closed-cycle helium refrigerator. Continuous-wave 474 nm laser and pulsed 405 nm laser were used as excitations for PL spectra/mapping and time-resolved PL measurements, respectively. Long-pass filters and tunable bandpass filters were used to select the peaks during the time-resolved PL measurements.
- Contact angle was measured on FTA1000B dynamic contact angle analyzer.
- XAS measurements were performed at I06 beamline at Diamond Light Source, UK. The samples were measured at ∼10^–9^ mbar. XPS measurements were performed at I09 beamline. The beam size was around 15 μm by 35 μm. The core level spectra of MoS_2_ were acquired at photon energy of 1 keV. Samples were heated at 80 °C under 10^–7^ mbar for 1 h to remove physisorbed species prior to loading into a measurement chamber (10^–10^ mbar). The Fermi level was calibrated by using a clean Au foil in contact with the sample holder. The spectral resolution was determined by measuring and fitting the Fermi edge of a Au foil with a Gaussian broadened Fermi–Dirac distribution and was determined to be 0.21 eV.
- XRD was measured on a Bruker D8 Advance powder X-ray diffractometer using Cu Kα radiation, equipped with automatic divergence slits and a LynxEye-XE position sensitive detector.

## Supporting Information Available

Schematic of wet transfer of exfoliated TMDs across various substrates; optical images of exfoliated pristine MoS_2_ before and after transfer from a SiO_2_/Si substrate to another SiO_2_/Si substrate; AFM images and PL spectra of transferred pristine monolayer MoS_2_ and postannealed pristine monolayer MoS_2_, respectively; synchrotron XPS spectra of the Mo 3d core level in pristine MoS_2_ on untreated SiO_2_ and on untreated HfO_2_; deconvoluted RT PL spectra of transferred defective monolayer MoS_2_ and pristine MoS_2_ on untreated and annealed SiO_2_, respectively; WCA measurement of (a) untreated/annealed SiO_2_ and HfO_2_; O *K*-edge XAS characterization of untreated/annealed SiO_2_ and HfO_2_; RT PL spectra of monolayer MoS_2_ annealed on HfO_2_; and XRD patterns of untreated and annealed HfO_2_ (PDF)

## References

[ref1] Bai G.; Yuan S.; Zhao Y.; Yang Z.; Choi S. Y.; Chai Y.; Yu S. F.; Lau S. P.; Hao J. 2D Layered Materials of Rare-Earth Er-Doped MoS_2_ with NIR-to-NIR Down- and Up-Conversion Photoluminescence. Adv. Mater. 2016, 28 (34), 7472–7477. 10.1002/adma.201601833. 27323249

[ref2] Zhang Q.; Wee A. T. S.; Liang Q.; Zhao X.; Liu M. Defect Engineering of Two-Dimensional Transition-Metal Dichalcogenides: Applications, Challenges, and Opportunities. ACS Nano 2021, 15 (2), 2165–2181. 10.1021/acsnano.0c09666. 33449623

[ref3] Yang J.; Wang Y.; Lagos M. J.; Manichev V.; Fullon R.; Song X.; Voiry D.; Chakraborty S.; Zhang W.; Batson P. E.; Feldman L.; Gustafsson T.; Chhowalla M. Single Atomic Vacancy Catalysis. ACS Nano 2019, 13 (9), 9958–9964. 10.1021/acsnano.9b05226. 31398001

[ref4] Li L.; Qin Z.; Ries L.; Hong S.; Michel T.; Yang J.; Salameh C.; Bechelany M.; Miele P.; Kaplan D.; Chhowalla M.; Voiry D. Role of Sulfur Vacancies and Undercoordinated Mo Regions in MoS_2_ Nanosheets toward the Evolution of Hydrogen. ACS Nano 2019, 13 (6), 6824–6834. 10.1021/acsnano.9b01583. 31136708

[ref5] Mitterreiter E.; Schuler B.; Micevic A.; Hernangómez-Pérez D.; Barthelmi K.; Cochrane K. A.; Kiemle J.; Sigger F.; Klein J.; Wong E.; Barnard E. S.; Watanabe K.; Taniguchi T.; Lorke M.; Jahnke F.; Finley J. J.; Schwartzberg A. M.; Qiu D. Y.; Refaely-Abramson S.; Holleitner A. W.; Weber-Bargioni A.; Kastl C. The Role of Chalcogen Vacancies for Atomic Defect Emission in MoS_2_. Nat. Commun. 2021, 12 (1), 3822 10.1038/s41467-021-24102-y. 34158488 PMC8219741

[ref6] Zhang Z.; Liang H.; Loh L.; Chen Y.; Chen Y.; Watanabe K.; Taniguchi T.; Quek S. Y.; Bosman M.; Bettiol A. A.; Eda G. Optically Active Chalcogen Vacancies in Monolayer Semiconductors. Adv. Opt. Mater. 2022, 10 (23), 2201350 10.1002/adom.202201350.

[ref7] Koperski M.; Nogajewski K.; Arora A.; Cherkez V.; Mallet P.; Veuillen J. Y.; Marcus J.; Kossacki P.; Potemski M. Single Photon Emitters in Exfoliated WSe_2_ Structures. Nat. Nanotechnol. 2015, 10 (6), 503–506. 10.1038/nnano.2015.67. 25938573

[ref8] Parto K.; Azzam S. I.; Banerjee K.; Moody G. Defect and Strain Engineering of Monolayer WSe_2_ Enables Site-Controlled Single-Photon Emission up to 150 K. Nat. Commun. 2021, 12 (1), 3585 10.1038/s41467-021-23709-5. 34117243 PMC8196156

[ref9] Li Z.; Nameirakpam H.; Berggren E.; Noumbe U.; Kimura T.; Asakura E.; Gray V.; Thakur D.; Edvinsson T.; Lindblad A.; Kohda M.; Araujo R. B.; Rao A.; Kamalakar M. V. Synchronized Photoluminescence and Electrical Mobility Enhancement in 2D WS_2_ through Sequence-Specific Chemical Passivation. J. Am. Chem. Soc. 2024, 146 (51), 35146–35154. 10.1021/jacs.4c11052. 39662959 PMC11673568

[ref10] Lin Z.; Liu W.; Tian S.; Zhu K.; Huang Y.; Yang Y. Thermal Expansion Coefficient of Few-Layer MoS_2_ Studied by Temperature-Dependent Raman Spectroscopy. Sci. Rep. 2021, 11 (1), 7037 10.1038/s41598-021-86479-6. 33782514 PMC8007611

[ref11] Lloyd D.; Liu X.; Christopher J. W.; Cantley L.; Wadehra A.; Kim B. L.; Goldberg B. B.; Swan A. K.; Bunch J. S. Band Gap Engineering with Ultralarge Biaxial Strains in Suspended Monolayer MoS_2_. Nano Lett. 2016, 16 (9), 5836–5841. 10.1021/acs.nanolett.6b02615. 27509768

[ref12] McCreary A.; Ghosh R.; Amani M.; Wang J.; Duerloo K.-A. N.; Sharma A.; Jarvis K.; Reed E. J.; Dongare A. M.; Banerjee S. K.; Terrones M.; Namburu R. R.; Dubey M. Effects of Uniaxial and Biaxial Strain on Few-Layered Terrace Structures of MoS_2_ Grown by Vapor Transport. ACS Nano 2016, 10 (3), 3186–3197. 10.1021/acsnano.5b04550. 26881920

[ref13] Ugeda M. M.; Bradley A. J.; Shi S.-F.; da Jornada F. H.; Zhang Y.; Qiu D. Y.; Ruan W.; Mo S.-K.; Hussain Z.; Shen Z.-X.; Wang F.; Louie S. G.; Crommie M. F. Giant Bandgap Renormalization and Excitonic Effects in a Monolayer Transition Metal Dichalcogenide Semiconductor. Nat. Mater. 2014, 13 (12), 1091–1095. 10.1038/nmat4061. 25173579

[ref14] Borghardt S.; Tu J.-S.; Winkler F.; Schubert J.; Zander W.; Leosson K.; Kardynał B. E. Engineering of Optical and Electronic Band Gaps in Transition Metal Dichalcogenide Monolayers through External Dielectric Screening. Phys. Rev. Mater. 2017, 1 (5), 054001 10.1103/PhysRevMaterials.1.054001.

[ref15] Raja A.; Chaves A.; Yu J.; Arefe G.; Hill H. M.; Rigosi A. F.; Berkelbach T. C.; Nagler P.; Schüller C.; Korn T.; Nuckolls C.; Hone J.; Brus L. E.; Heinz T. F.; Reichman D. R.; Chernikov A. Coulomb Engineering of the Bandgap and Excitons in Two-Dimensional Materials. Nat. Commun. 2017, 8 (1), 15251 10.1038/ncomms15251. 28469178 PMC5418602

[ref16] Ji E.; Yang K.; Shin J.-C.; Kim Y.; Park J.-W.; Kim J.; Lee G.-H. Exciton-Dominant Photoluminescence of MoS_2_ by a Functionalized Substrate. Nanoscale 2022, 14 (38), 14106–14112. 10.1039/D2NR03455G. 36070461

[ref17] Nur R.; Tsuchiya T.; Toprasertpong K.; Terabe K.; Takagi S.; Takenaka M. High Responsivity in MoS_2_ Phototransistors Based on Charge Trapping HfO_2_ Dielectrics. Commun. Mater. 2020, 1 (1), 103 10.1038/s43246-020-00103-0.

[ref18] Li Y.; Steuer O.; Lin K.; Samad F.; Sokolova D.; Erbe A.; Helm M.; Zhou S.; Prucnal S. Influence of Dielectric Capping on the Optical Properties of Two-Dimensional Transition-Metal Dichalcogenides: Implications for Nano-Optoelectronics. ACS Appl. Opt. Mater. 2023, 1 (10), 1733–1741. 10.1021/acsaom.3c00296.

[ref19] Tran T. T.; Elbadawi C.; Totonjian D.; Lobo C. J.; Grosso G.; Moon H.; Englund D. R.; Ford M. J.; Aharonovich I.; Toth M. Robust Multicolor Single Photon Emission from Point Defects in Hexagonal Boron Nitride. ACS Nano 2016, 10 (8), 7331–7338. 10.1021/acsnano.6b03602. 27399936

[ref20] Munson K. T.; Torsi R.; Mathela S.; Feidler M. A.; Lin Y. C.; Robinson J. A.; Asbury J. B. Influence of Substrate-Induced Charge Doping on Defect-Related Excitonic Emission in Monolayer MoS_2_. J. Phys. Chem. Lett. 2024, 15 (31), 7850–7856. 10.1021/acs.jpclett.4c01578. 39052863

[ref21] Kim G.; Kim H. M.; Kumar P.; Rahaman M.; Stevens C. E.; Jeon J.; Jo K.; Kim K.-H.; Trainor N.; Zhu H.; Sohn B.-H.; Stach E. A.; Hendrickson J. R.; Glavin N. R.; Suh J.; Redwing J. M.; Jariwala D. High-Density, Localized Quantum Emitters in Strained 2D Semiconductors. ACS Nano 2022, 16 (6), 9651–9659. 10.1021/acsnano.2c02974. 35621266

[ref22] Zhu Y.; Lim J.; Zhang Z.; Wang Y.; Sarkar S.; Ramsden H.; Li Y.; Yan H.; Phuyal D.; Gauriot N.; Rao A.; Hoye R. L. Z.; Eda G.; Chhowalla M. Room-Temperature Photoluminescence Mediated by Sulfur Vacancies in 2D Molybdenum Disulfide. ACS Nano 2023, 17 (14), 13545–13553. 10.1021/acsnano.3c02103. 37418552 PMC10373523

[ref23] Sarkar S.; Goswami S.; Trushin M.; Saha S.; Panahandeh-Fard M.; Prakash S.; Tan S. J. R.; Scott M.; Loh K. P.; Adam S.; Mathew S.; Venkatesan T. Polaronic Trions at the MoS_2_/SrTiO_3_ Interface. Adv. Mater. 2019, 31 (41), 1–9. 10.1002/adma.201903569. 31448503

[ref24] Chakraborty B.; Bera A.; Muthu D. V. S.; Bhowmick S.; Waghmare U. V.; Sood A. K. Symmetry-Dependent Phonon Renormalization in Monolayer MoS_2_ Transistor. Phys. Rev. B 2012, 85 (16), 161403 10.1103/PhysRevB.85.161403.

[ref25] Ryou J.; Kim Y.-S.; KC S.; Cho K. Monolayer MoS_2_ Bandgap Modulation by Dielectric Environments and Tunable Bandgap Transistors. Sci. Rep. 2016, 6 (1), 29184 10.1038/srep29184. 27378032 PMC4932597

[ref26] Komsa H. P.; Krasheninnikov A. V. Effects of Confinement and Environment on the Electronic Structure and Exciton Binding Energy of MoS_2_ from First Principles. Phys. Rev. B: Condens. Matter Mater. Phys. 2012, 86 (24), 241201 10.1103/PhysRevB.86.241201.

[ref27] Naik M. H.; Jain M. Substrate Screening Effects on the Quasiparticle Band Gap and Defect Charge Transition Levels in MoS_2_. Phys. Rev. Mater. 2018, 2 (8), 084002 10.1103/PhysRevMaterials.2.084002.

[ref28] Mak K. F.; He K.; Lee C.; Lee G. H.; Hone J.; Heinz T. F.; Shan J. Tightly Bound Trions in Monolayer MoS_2_. Nat. Mater. 2013, 12 (3), 207–211. 10.1038/nmat3505. 23202371

[ref29] McClellan C. J.; Yalon E.; Smithe K. K. H.; Suryavanshi S. V.; Pop E. High Current Density in Monolayer MoS _2_ Doped by AlO*_x_*. ACS Nano 2021, 15 (1), 1587–1596. 10.1021/acsnano.0c09078. 33405894

[ref30] Park K.; Kang H.; Koo S.; Lee D.; Ryu S. Redox-Governed Charge Doping Dictated by Interfacial Diffusion in Two-Dimensional Materials. Nat. Commun. 2019, 10 (1), 4931 10.1038/s41467-019-12819-w. 31666518 PMC6821894

[ref31] Khan S. B.; Zhang Z.; Lee S. L. Annealing Influence on Optical Performance of HfO_2_ Thin Films. J. Alloys. Compd. 2020, 816, 152552 10.1016/j.jallcom.2019.152552.

[ref32] Shim J.; Rivera J. A.; Bashir R. Electron Beam Induced Local Crystallization of HfO_2_ Nanopores for Biosensing Applications. Nanoscale 2013, 5 (22), 10887–10893. 10.1039/c3nr02608f. 23945603 PMC3867606

[ref33] Ni J.; Zhou Q.; Li Z.; Zhang Z. Oxygen Defect Induced Photoluminescence of HfO_2_ Thin Films. Appl. Phys. Lett. 2008, 93 (1), 11905 10.1063/1.2952288.

[ref34] Tongay S.; Suh J.; Ataca C.; Fan W.; Luce A.; Kang J. S.; Liu J.; Ko C.; Raghunathanan R.; Zhou J.; Ogletree F.; Li J.; Grossman J. C.; Wu J. Defects Activated Photoluminescence in Two-Dimensional Semiconductors: Interplay between Bound, Charged, and Free Excitons. Sci. Rep. 2013, 3, 1–5. 10.1038/srep02657. PMC3772378 24029823

[ref35] Buscema M.; Steele G. A.; van der Zant H. S. J.; Castellanos-Gomez A. The Effect of the Substrate on the Raman and Photoluminescence Emission of Single-Layer MoS_2_. Nano Res. 2014, 7 (4), 561–571. 10.1007/s12274-014-0424-0.

[ref36] Hötger A.; Klein J.; Barthelmi K.; Sigl L.; Sigger F.; Männer W.; Gyger S.; Florian M.; Lorke M.; Jahnke F.; Taniguchi T.; Watanabe K.; Jöns K. D.; Wurstbauer U.; Kastl C.; Müller K.; Finley J. J.; Holleitner A. W. Gate-Switchable Arrays of Quantum Light Emitters in Contacted Monolayer MoS_2_ Van Der Waals Heterodevices. Nano Lett. 2021, 21 (2), 1040–1046. 10.1021/acs.nanolett.0c04222. 33433221

[ref37] Ross J. S.; Wu S.; Yu H.; Ghimire N. J.; Jones A. M.; Aivazian G.; Yan J.; Mandrus D. G.; Xiao D.; Yao W.; Xu X. Electrical Control of Neutral and Charged Excitons in A Monolayer Semiconductor. Nat. Commun. 2013, 4 (1), 1–6. 10.1038/ncomms2498. 23403575

[ref38] Lien D.-H.; Uddin S. Z.; Yeh M.; Amani M.; Kim H.; Ager J. W.; Yablonovitch E.; Javey A. Electrical Suppression of All Nonradiative Recombination Pathways in Monolayer Semiconductors. Science 2019, 364 (6439), 468–471. 10.1126/science.aaw8053. 31048488

[ref39] Gieraltowska S.; Wachnicki L.; Dluzewski P.; Witkowski B. S.; Godlewski M.; Guziewicz E. Atomic Layer Deposition of HfO_2_ Films Using TDMAH and Water or Ammonia Water. Materials 2023, 16 (11), 4077 10.3390/ma16114077. 37297215 PMC10254648

