# Supplementary material for: Effect of Substrate on Sulfur Vacancy Defect-Mediated Photoluminescence in Two-Dimensional MoS2
Source: J Phys Chem C Nanomater Interfaces. 2025 Apr 18;129(17):8294–302. doi: 10.1021/acs.jpcc.4c08491 (PMC12051199; doi:10.1021/acs.jpcc.4c08491)
Supplement: Supplementary file 1 — jp4c08491_si_001.pdf [file jp4c08491_si_001.pdf]

Supporting Information

for

Effect of substrate on sulfur vacancy defect mediated photoluminescence in two-dimensional MoS_2

Yiru Zhu[1], Zhepeng Zhang[2], Ye Wang[1], Soumya Sarkar[1], Yang Li[1], Han Yan[1], Larissa Ishibe-Veiga[3], Anita Bagri[3], Ziwei Jeffrey Yang[1], Hugh Ramsden[1], Goki Eda[2,4,5], Robert L.Z. Hoye[6], Yan Wang[1], Manish Chhowalla[1*]*

1 Department of Materials Science and Metallurgy, University of Cambridge, 27 Charles Babbage Rd, Cambridge CB3 0FS, UK.

2 Department of Physics, National University of Singapore, 2 Science Drive 3, Singapore 117551.

3 Diamond Light Source, Chilton, Didcot, Oxfordshire, OX11 0DE UK.

4 Department of Chemistry, National University of Singapore, 3 Science Drive 3, 117543, Singapore.

5 Centre for Advanced 2D Materials, National University of Singapore, 6 Science Drive 2, 117542, Singapore.

6 Inorganic Chemistry Laboratory, University of Oxford, South Parks Road, Oxford OX1 3QR, UK.

We found that the MoS_2 adhered strongly to SiO_2 after annealing. Because of this, we adopted the poly(bisphenol A carbonate) (PC) transfer method, as shown in **Figure S1**. This transfer method was tested by transferring pristine MoS_2 from the original SiO_2/Si substrate to another SiO_2/Si substrate. The transfer method was optimized by monitoring changes in morphology and PL, as shown in **Figures S2,S3**.

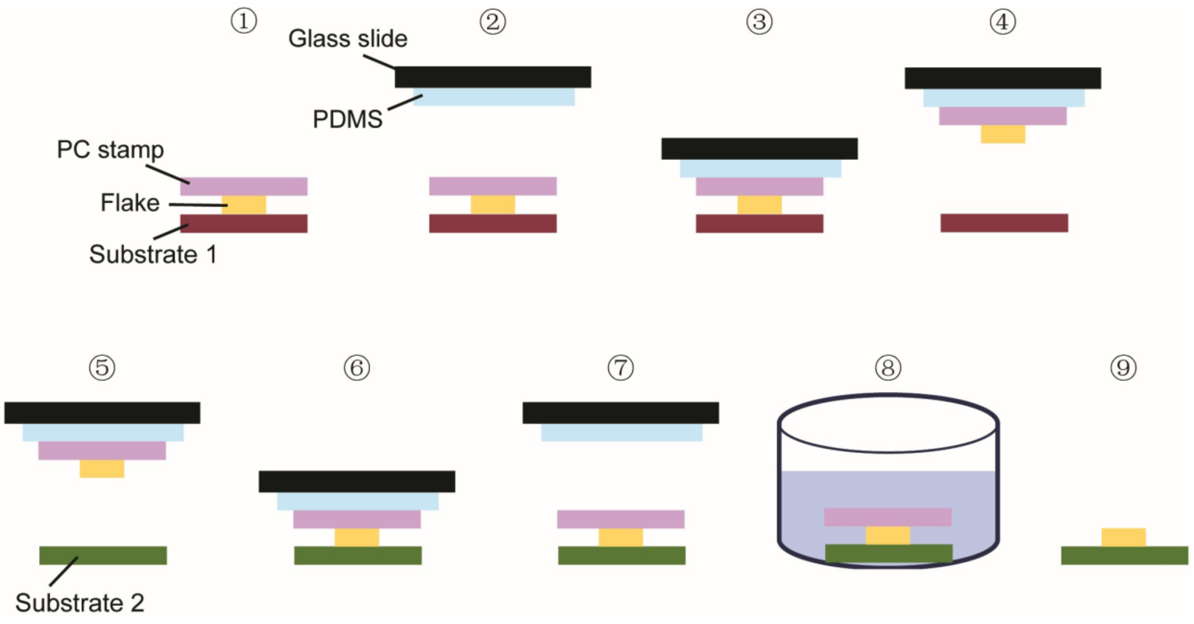

Figure S1. Schematic of wet transfer of exfoliated TMDs across various substrates. ① PC stamp is drop-cast on the exfoliated sample. ② A PDMS stamp stacked on a glass slide is placed face-down. ③ The PDMS stamp is brought in contact with the PC stamp to form a firm PDMS/PC stack. ④ The target flake is picked up by the PDMS/PC stack from substrate 1. ⑤ The target flake is aligned with substrate 2. ⑥ The PDMS/PC stack is brought in contact with substrate 2. ⑦ The PDMS stamp is raised up to detach from the PC stamp. ⑧ The sample is immersed in chloroform to dissolve the PC stamp. ⑨ The target flake is left on substrate 2.

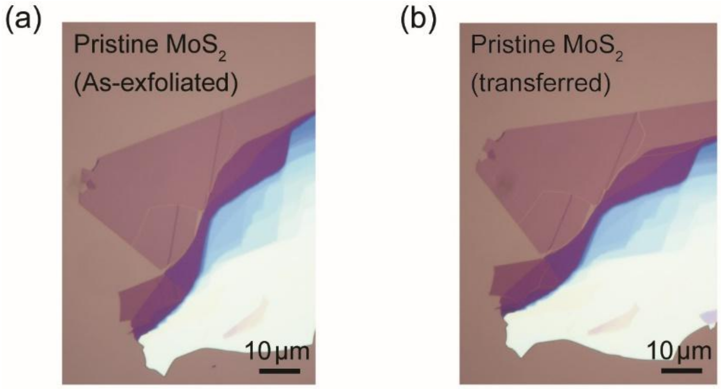

Figure S2. Optical microscopy images of exfoliated pristine MoS_2 (a) before and (b) after transfer from a SiO_2/Si substrate to another SiO_2/Si substrate. The transferred flake maintains the high quality, without new wrinkles or cracks.

The morphology of the transferred flakes was characterized by AFM. On the transferred monolayer MoS_2, PC residues were observed across the whole flake (**Figure S3a**). The PL features of monolayer TMDs are sensitive to extrinsic contaminants that can be identified using the ratio of the integrated intensity of A^- trion to A exciton, $I(A^-)/I(A)$. This ratio increases from 0.47 ± 0.05 in the as-exfoliated sample to 0.84 ± 0.09 in the transferred sample, shown in **Figure S3b,c**. To remove the PC residues, the transferred sample was post-annealed in Ar/H_2 (95 *vol*.%/5 *vol*.%) at 350 °C for 1 h. AFM image in **Figure S3d** shows that the sample was cleaned by post-annealing with the $I(A^-)/I(A)$ ratio restoring to 0.44 ± 0.13 – comparable to the as-exfoliated pristine MoS_2 samples, shown in **Figure S3e,f**.

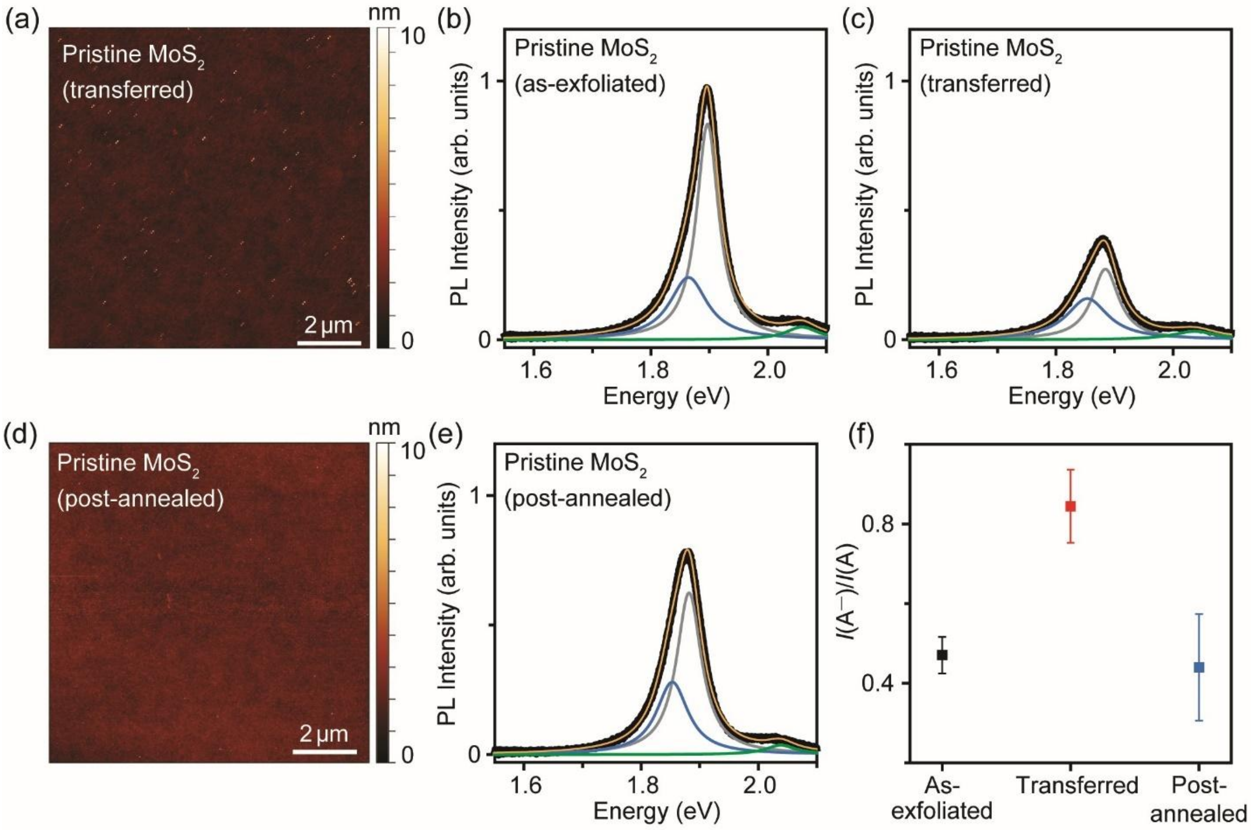

Figure S3. Transfer and post-annealing of pristine MoS_2. (a,d) AFM image of transferred and post-annealed pristine monolayer MoS_2. The transferred sample shows PC residues across the whole flake while post-annealed sample shows a clean surface without noticeable residues. (b,c,e) PL spectra of pristine MoS_2 (b) before and (c) after transfer and (e) after post-annealing fitted with Lorentz peaks. Denotation: A exciton in grey, A^- trion in blue, B exciton in green, and cumulative fit curve in yellow. (f) The ratio of the integrated intensity of A^- trion to A exciton, $I(A^-)/I(A)$, for pristine, transferred, and post-annealed MoS_2. The ratio is increased by transferring the flake and restored by post-annealing.

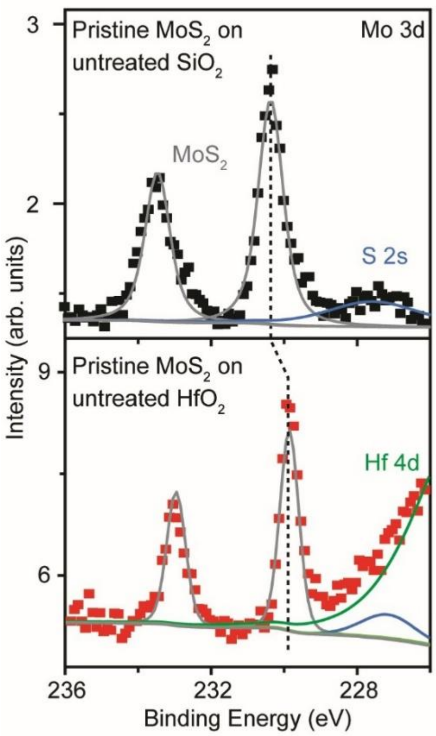

Figure S4. Synchrotron XPS spectra of the Mo 3d core level in pristine MoS_2 on untreated SiO_2 and on untreated HfO_2 measured using synchrotron 1 keV soft X-rays. MoS_2 on untreated SiO_2 shows the characteristic Mo 3d doublet from stoichiometric MoS_2 (denoted in grey) and S 2s peak (denoted in blue). MoS_2 on untreated HfO_2 shows additional Hf 4d doublet (denoted in green), with Mo 3d doublet and S 2s peak showing lower binding energy than pristine MoS_2/SiO_2, indicating that MoS_2 is less electron-doped by HfO_2.

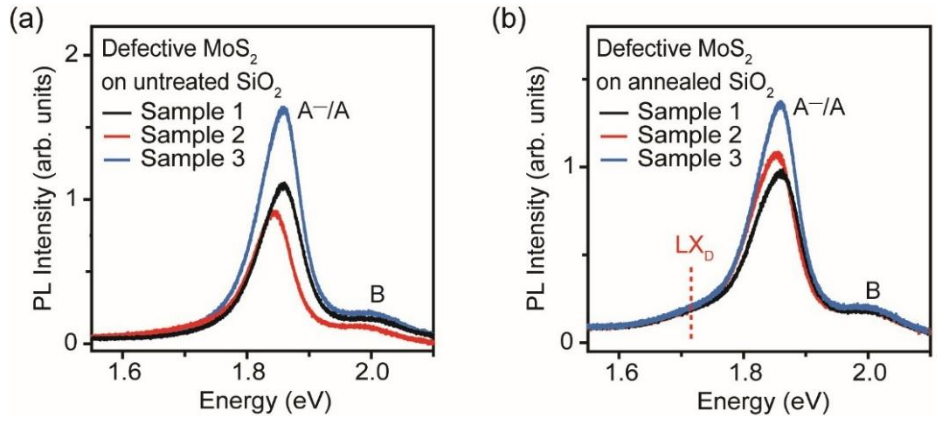

Figure S5. RT PL spectra of defective monolayer MoS_2 on (a) untreated and (b) annealed SiO_2. LX_D emission is observed in defective MoS_2 on annealed SiO_2 but not on untreated SiO_2. Each set contains three samples.

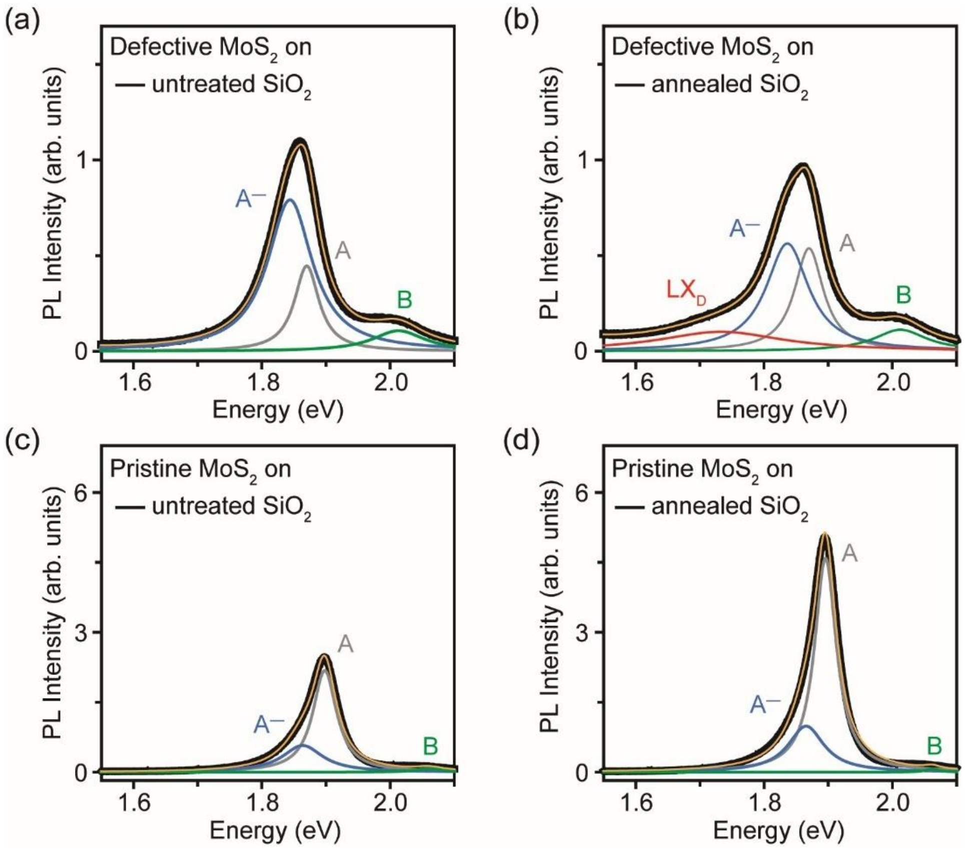

Figure S6. Deconvoluted RT PL spectra of defective and pristine monolayer MoS_2 on untreated and annealed SiO_2, fitted with Lorentz peaks. Denotation: A exciton in grey, A^- trion in blue, B exciton in green, LX_D emission in red, and cumulative fit curve in yellow. (a–b) RT PL spectra of defective MoS_2 on (a) untreated and (b) annealed SiO_2. PL in (a) consists of A exciton, A^- trion, and B exciton only, while in (b) it shows an additional LX_D peak. For defective MoS_2, the ratio of integrated intensity of A^- trion to A exciton, $I(A^-)/I(A)$, is higher on untreated SiO_2 (3.22) than on annealed SiO_2 (1.54). (c–d) RT PL spectra of pristine MoS_2 on (c) untreated and (d) annealed SiO_2. For pristine MoS_2, the $I(A^-)/I(A)$ ratio, is higher on untreated SiO_2 (0.44) than on annealed SiO_2 (0.35).

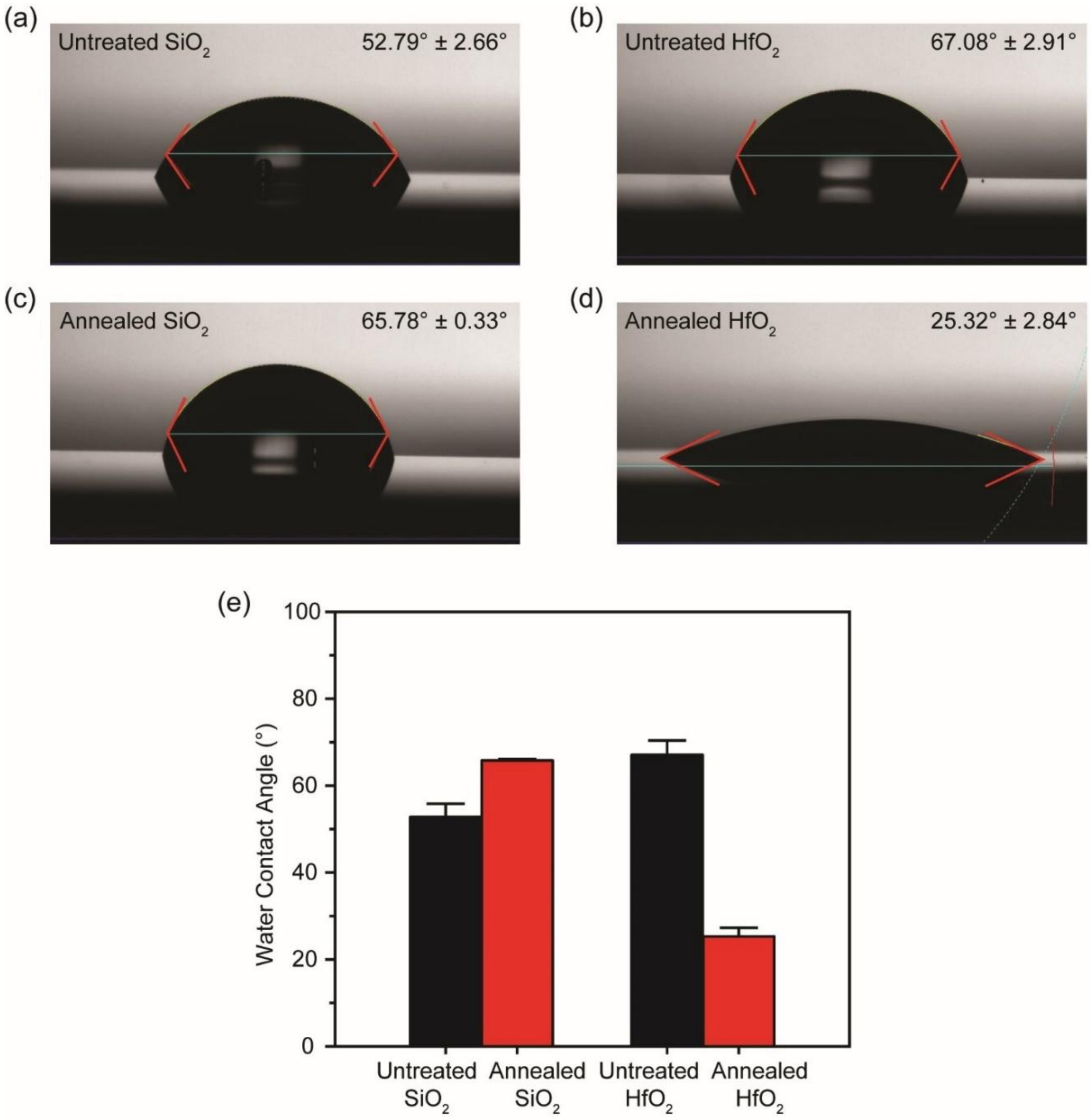

Figure S7. Water contact angle (WCA) measurement of (a) untreated SiO_2, (b) annealed SiO_2, (c) untreated HfO_2, and (d) annealed HfO_2. (e) Plot of the WCA measured on untreated/annealed SiO_2 and HfO_2, respectively, showing larger water contact angle (less hydrophilic) on annealed SiO_2 than on untreated SiO_2 while lower water contact angle (more hydrophilic) on annealed HfO_2 than on untreated HfO_2. The error bars are calculated from 3 points per substrate.

The chemical nature of untreated/annealed SiO_2 and HfO_2 was characterized by synchrotron XAS (**Figure S7**). The oxide surface was encapsulated by MoS_2 to minimize the influence of the ambient environment. Using X-ray energies > 530 eV, the oxygen *K*-edge was measured by exciting an O 1s core electron to an empty 2p state (**Figure S7a**). The spectrum consists of three

regions: (a) the O *K* pre-edge: < E_0 (E_0 is the first maximum on the first derivative curve) which is ~536 eV for SiO_2 and 532 eV for HfO_2; (b) the O *K* edge: E_0 to 575 eV; and (c) the O K post-edge: 575 to 580 eV.[1] The intensities of the spectrum were normalized to the pre-edge/post-edge to generate a normalized XAS spectrum going from 0 below the absorption edge to 1 above the edge). This is to regularize the XAS spectra with respect to variations in sample and measurement so that the normalized data can be directly compared. For untreated SiO_2, the three primary features at 537.6 eV, 547.2 eV, and 560 eV correspond to hybridized O 2p-Si 3s state, O 2p-Si 3p and O 2p-Si 3d state transitions, respectively, as shown in **Figure 3b,d**.[1] Compared to pristine SiO_2, the overall O *K*-edge signals in annealed SiO_2 are enhanced, suggesting higher absorption of X-rays – possibly due to stronger coupling between O and Si orbitals caused by the formation of hydrophobic siloxane (Si–O–Si).[2] The features at ~535–536 eV may originate from oxygen deficiencies in SiO_2 that introduces in-gap features.[3] Compared to pristine SiO_2, the decrease in E_0 by 0.4 eV in annealed SiO_2 suggests a decrease in the O valence state.[2] We think this might be due to the formation of Si–O[4] or the reduced electron transfer from annealed SiO_2 to MoS_2[5].

The XAS spectra of HfO_2 show two main features at approximately 533 and 537 eV, respectively, corresponding to transitions to O 2p orbitals that are hybridized with Hf 5d e_g and t_{2g} states, as shown in **Figure 3c,d**. The spectrum from the annealed HfO_2 films shows three additional well-defined peaks at 541.6, 543.9, and 549.7 eV at the higher energy region, associated with transitions to O 2p levels hybridized with Hf 6s and 6p states. The appearance of these three peaks has been identified as a signature of crystalline HfO_2.[6] The crystallization of HfO_2 due to Ar/H_2 annealing was corroborated by X-ray diffraction (XRD), shown in SI, **Figure S9**. The untreated HfO_2 only shows signals from underlying crystalline Si (111) because it is amorphous. The Ar/H_2 annealed HfO_2 shows additional peaks that are attributed to the monoclinic phase of crystalline HfO_2.[7] Correspondingly, we also observed the Hf 5d e_g-related feature of the annealed HfO_2 films splitting into two components at 532.0 and 532.9 eV as a result of the crystal field effect. Compared to pristine HfO_2, the E_0 in annealed HfO_2 increases by 0.3 eV, indicating an increase in the O valence state that could be due to the crystallization of HfO_2 which redistributes the surface charges or enhances electron transfer from annealed HfO_2 to MoS_2 – opposite to the case for SiO_2.

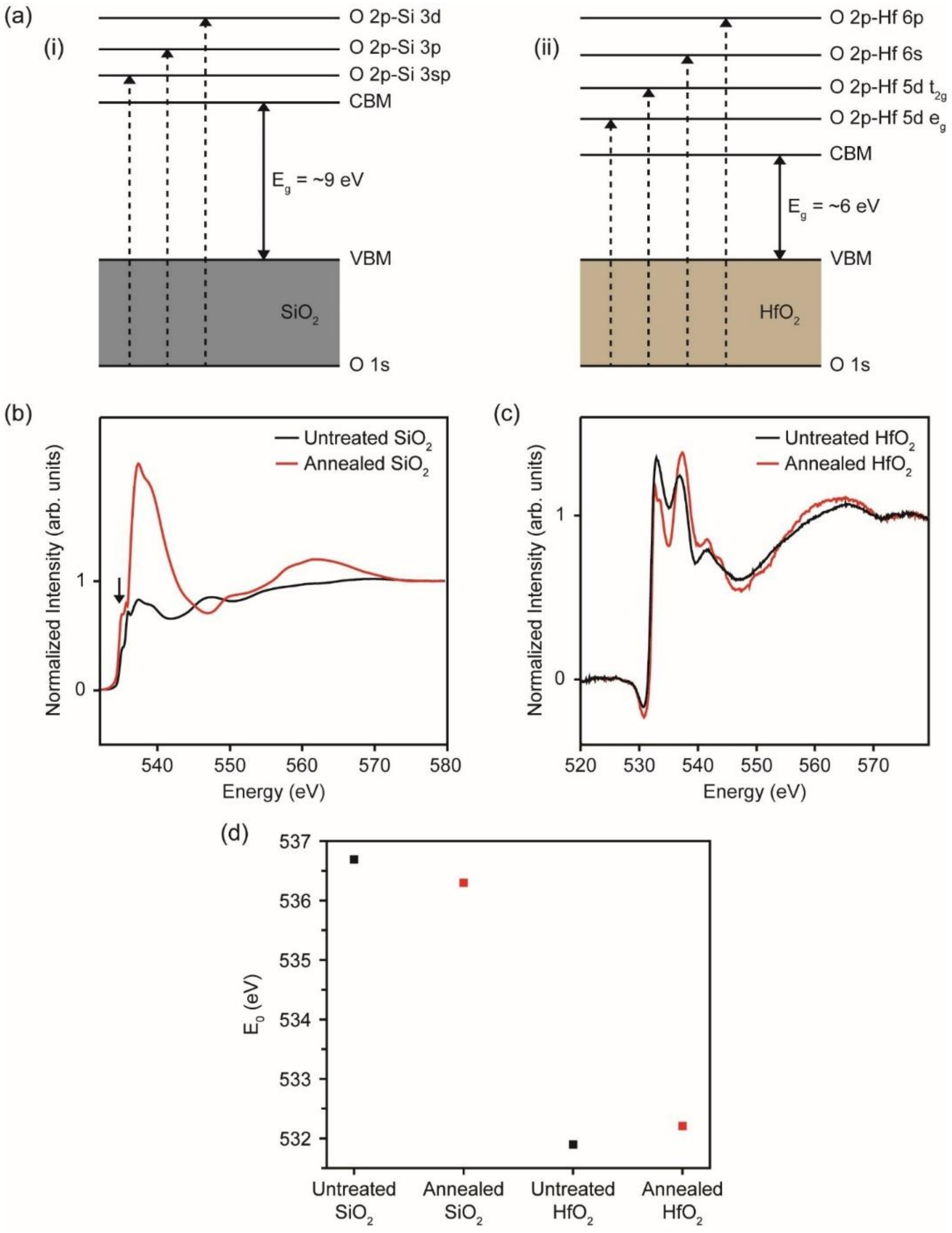

Figure S8. O *K*-edge XAS characterization of untreated/annealed SiO_2 and HfO_2. (a) Schematic of O *K*-edge XAS measurement of (i) SiO_2 and (ii) HfO_2, showing the excitation of O 1s state to O 2p state. (b–c) XAS spectra of (b) untreated/annealed SiO_2, and (c) untreated/annealed HfO_2.

Annealed SiO_2 shows stronger overall O *K*-edge signals, suggesting stronger coupling between O and Si. Annealed HfO_2 shows clear splitting of O 2p–Hf 5d e_g states, attributed to crystal field effect. (d) Plot of edge onset E_0 of untreated/annealed SiO_2 and HfO_2, taken from the first derivative of XAS spectra in (c–d). Annealed SiO_2 shows a smaller E_0 than untreated SiO_2, while annealed HfO_2 shows a higher E_0 than untreated HfO_2, implying the reduced electron transfer from annealed SiO_2 to MoS_2 and the increased electron transfer from annealed HfO_2 to MoS_2.

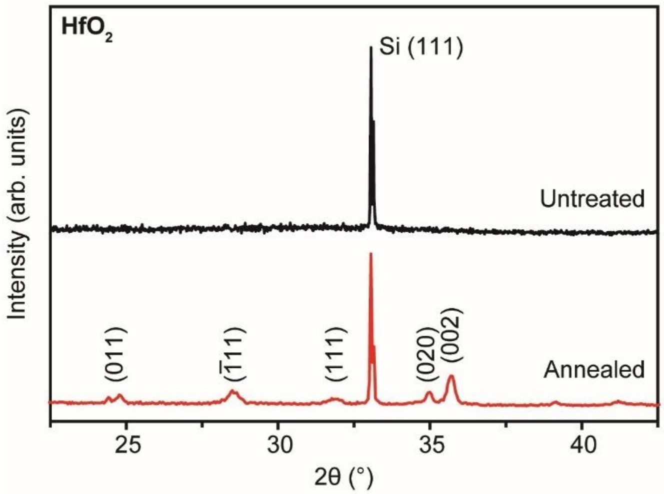

Figure S9. XRD patterns of untreated and annealed HfO_2. The untreated HfO_2 shows sharp peaks of Si (111) from the underlying Si substrate. The annealed HfO_2 shows additional peaks that are attributed to the monoclinic phase of crystalline HfO_2.

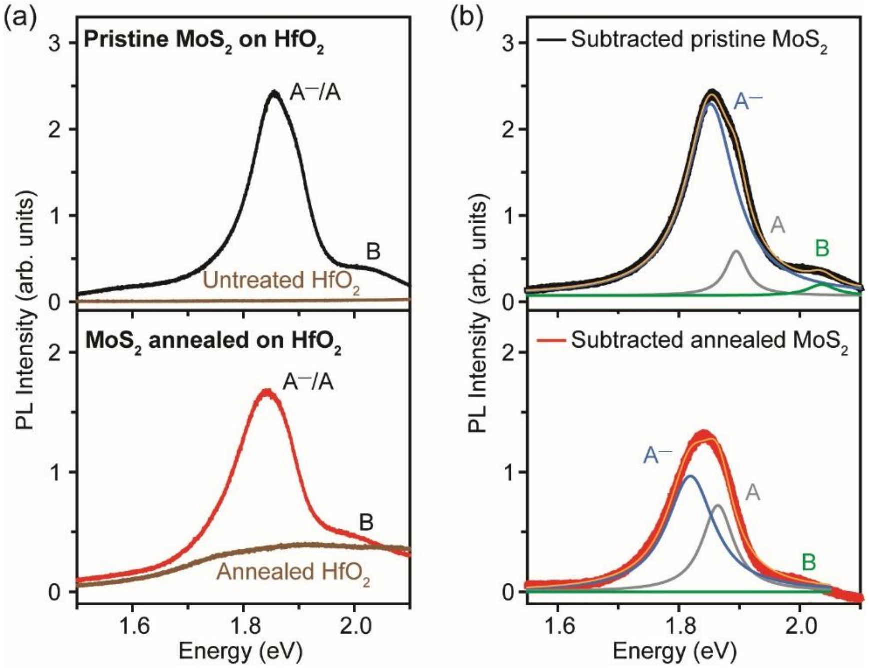

Figure S10. RT PL spectra of monolayer MoS_2 annealed on HfO_2. (a) RT PL spectra of pristine MoS_2 on HfO_2 (top panel) and annealed MoS_2 (bottom panel). The background PL signals from HfO_2 substrate are shown in brown. The broad PL feature in annealed HfO_2 is attributed to oxygen vacancies.[8] (b) Background-subtracted RT PL spectra of pristine and annealed MoS_2, deconvoluted with Lorentz peaks. Denotation: A exciton in grey, A^- trion in blue, B exciton in green, and cumulative fit curve in yellow. Only the A^-/A peak and B exciton peaks are present in both pristine and annealed MoS_2.

REFERENCES

(1) Frati, F.; Hunault, M. O. J. Y.; De Groot, F. M. F. Oxygen K-Edge X-Ray Absorption Spectra. *Chem. Rev.* **2020**, *120* (9), 4056–4110.

(2) Suntivich, J.; Hong, W. T.; Lee, Y. L.; Rondinelli, J. M.; Yang, W.; Goodenough, J. B.; Dabrowski, B.; Freeland, J. W.; Shao-Horn, Y. Estimating Hybridization of Transition Metal and Oxygen States in Perovskites from O K-Edge X-Ray Absorption Spectroscopy. *J. Phys. Chem. C* **2014**, *118* (4), 1856–1863.

(3) Cho, D. Y.; Jung, H. S.; Yu, I. H.; Park, W. G.; Cho, S.; Kim, U.; Oh, S. J.; Park, B. G.; Chang, F. H.; Lin, H. J.; Hwang, C. S. Nondestructive Investigation of Interface States in

High-k Oxide Films on Ge Substrate Using X-Ray Absorption Spectroscopy. *Phys. Status Solidi - Rapid Res. Lett.* **2012**, *6* (4), 181–183.

(4) Paparazzo, E.; Fanfoni, M.; Severini, E.; Priori, S. Evidence of Si–OH Species at the Surface of Aged Silica. *Journal of Vacuum Science & Technology A: Vacuum, Surfaces, and Films* **1992**, *10* (4), 2892–2896.

(5) Li, Y.; Hou, P.; Xi, Z.; Xu, Y.; Liu, Y.; Tian, H.; Li, J.; Yang, Y.; Deng, Y.; Wu, D. Charge Transfer Driving Interfacial Reconstructions in Perovskite Oxide Heterostructures. *Commun. Phys.* **2023**, *6* (1), 70.

(6) Hill, D. H.; Bartynski, R. A.; Nguyen, N. V.; Davydov, A. C.; Chandler-Horowitz, D.; Frank, M. M. The Relationship between Local Order, Long Range Order, and Sub-Band-Gap Defects in Hafnium Oxide and Hafnium Silicate Films. *J. Appl. Phys.* **2008**, *103* (9), 93712.

(7) Ho, M.-Y.; Gong, H.; Wilk, G. D.; Busch, B. W.; Green, M. L.; Voyles, P. M.; Muller, D. A.; Bude, M.; Lin, W. H.; See, A.; Loomans, M. E.; Lahiri, S. K.; Räisänen, P. I. Morphology and Crystallization Kinetics in HfO_2 Thin Films Grown by Atomic Layer Deposition. *J. Appl. Phys.* **2003**, *93* (3), 1477–1481.

(8) Ni, J.; Zhou, Q.; Li, Z.; Zhang, Z. Oxygen Defect Induced Photoluminescence of HfO_2 Thin Films. *Appl. Phys. Lett.* **2008**, *93* (1), 11905.
